# Combined presence of coagulation factor XIII V34L and plasminogen activator inhibitor 1 4G/5G gene polymorphisms significantly contribute to recurrent pregnancy loss in Serbian population

**DOI:** 10.2478/jomb-2019-0028

**Published:** 2020-01-23

**Authors:** Ivana Joksic, Zeljko Mikovic, Dejan Filimonovic, Jelena Munjas, Orlic Natasa Karadzov, Amira Egic, Gordana Joksic

**Affiliations:** 1 Gynecology and Obstetrics Clinic 'Narodni front', Genetic laboratory department, Belgrade; 2 Gynecology and Obstetrics Clinic 'Narodni front', High-risk pregnancy department, Belgrade; 3 University of Belgrade, Faculty of Pharmacy, Departement of Medical Biochemistry, Belgrade

**Keywords:** factor XIII, gene polymorphism, inherited thrombophilia, plasminogen activator inhibitor-1, recurrent pregnancy loss, ponavljani spontani pobačaji, nasledne trombofilije polimorfizam, inhibitor aktivatora plazminogena-1, faktor XIII

## Abstract

**Background:**

Recurrent pregnancy loss (RPL) is a heterogeneous condition affecting up to 5% of women of reproductive age. Inherited thrombophilia have been postulated as one of the causes of RPL. Here we examined the prevalence of nine thrombophilic gene polymorphisms among women with history of recurrent miscarriages and fertile controls.

**Methods:**

The study included 70 women with history of at least three early pregnancy losses and 31 fertile controls with no miscarriages. We investigated mutations in genes responsible for clotting and fibrinolysis, including factor V (FV) Leiden, FV H1299R, factor II (FII) G20210A, methylene tetrahydrofolate reductase (MTHFR) C677T and A1298C, factor XIII (FXIII) V34L, plasminogen activator inhibitor-1 (PAI-1) 4G/5G and endothelial protein C receptor (EPCR) H1 and H3 haplotypes using reverse polymerase chain reaction ViennaLab cardiovascular disease StrippAssays.

**Results:**

Our results showed no significant increase in prevalence of tested polymorphisms in women with RPL. However, relative risk for PRL among women heterozygous for FXIII V34L was 2.81 times increased (OR 2.81, 95% CI 1.15-6.87, P=0.023). Haplotype analysis showed that combined presence of high-risk genotypes for FXIII and PAI-1 significantly increases risk for RPL (OR 13.98, CI 95% 1.11-17.46, P=0.044).

**Conclusions:**

This is the first study in Serbian population that investigated prevalence of FVR2, A1298C, FXIII V34L and EPCR gene variants. Compound heterozygosity for FXIII V34L and PAI-1 4G is significant risk factor for recurrent miscarriage. Our results should be viewed in context of small case-control study, so further large prospective studies are need for confirmation of our findings.

## Abstract (sr)

**Uvod:**

Ponavljani spontani pobačaji (PSP) su etiološki heterogeni i javljaju se kod 5% parova u reproduktivnom period. Jedan od mogućih uzroka PSP su i nasledne trombofilije. U okviru ove studije analizirali smo učestalost devet trombofilnih polimorfizama kod pacijentkinja sa ponavljanim spontanim pobačajima.

**Metode:**

Ispitanici su u studiji podeljeni u dve grupe na osnovu anamnestičkih podataka o broju spontanih pobačaja (70 u grupi sa PSP i 31 u kontrolnoj grupi). Ispitivani su sledeći genski polimorfizmi: faktor V Lajden (FVL), FVR2, faktor II (FII) G21210A, metilentetrahidrofolat reduktaza (MTHFR) C677T i A1298C polimorfizmi, inhibitor aktivatora plazminogena 1 (PAI-1) 4G/5G, faktor XIII (FXIII) V34L i endotelni protein C receptor (EPRC) H1, H2 i H3 haplotipovi. Za detekciju navedenih polimorfizama je korišćena metoda multipleks reakcije lančanog umnožavanja i reverzne hibridizacije na ViennaLab stripovima.

**Rezultati:**

Dobijeni rezultati nisu pokazali povećanu učestalost ispitivanih polimorfizama u grupi sa PSP. Posmatrajući uticaj pojedinačnih polimorfizama na ishod trudnoće pokazano je da polimorfizam FXIII V34L povećava rizik za ponavljane spontane pobačaje (OR 2,81, 95%CI 1,15-6,87, P=0,023). Analizom haplotipova ustanovljeno je da kombinovano prisustvo V34L i PAI-1 4G varijanti značajno povećava rizik za PSP (OR 13,98, CI 95% 1,11-17,46, P=0,044).

**Zaključak:**

Ovo je prva studija koja je ispitivala prevalencu FVR2, A1298C, FXIII V34L and EPCR polimorfizama u populaciji žena iz Srbije. Složeni heterozigoti za FXIII V34L i PAI-1 4G polimorfizme imaju značajno povišen rizik sa ponavljane gubitke trudnoće. Radi potvrde dobijenih rezultata potrebne su veće prospektivne studije.

## Introduction

Recurrent pregnancy loss (RPL) is heterogeneous condition affecting up to 5% of couples [1] [2]. It is defined as three or more consecutive miscarriages [3]. Numerous factors such as chromosomal aberrations, endocrinological, infective and immunologic diseases, anatomic abnormalities of the uterus or hypercoagulable states can cause RPL, but in more than 40% of cases etiology will remain unclear [4]. Inherited thrombophilia represents genetic predisposition for improper formation of blood clots, and it is caused by different sequence variants in genes coding for coagulation factors and enzymes included in fibrinolysis [5] [6]. Proper placental formation is necessary for successful pregnancy outcome. Several studies have proposed association between adverse pregnancy outcome and inherited thrombophilia, as it is shown that they can induce placental insufficiency due to vascular thrombosis [3] [7].

Gene variants in factor V gene (FV), prothrombin (FII), methylene tetrahydrofolate reductase (MTHFR) and plasminogen activator inhibitor-1 (PAI-1) are the most extensively studied in association with RPL [3] [4] [7]. FV Leiden (G1619A) abolishes cleavage site for activated protein C (APC), resulting in 10 times slower inactivation of FV [8]. Risk for RPL in pregnant women heterozygous carriers of FV Leiden is increased 2-3 times [9] [10]. Second gene polymorphism in FV, FVR2 (A4070G), combined with FVL, further increases risk for thrombosis [11]. However, FVR2 alone does not seem to significantly increase risk for venous thrombosis [11]. Prothrombin gene variant G20210A increases protein synthesis and also the odds ratio for RPL (OR 2-9) [12] [13] [14]. MTHFR C677T and A1298C variants mildly diminish enzyme activity, and if present with low serum folate levels represent risk factor for hiperhomocysteinemia [15] [16] [17] [18]. High serum levels of homocysteine are associated with numerous pathologic states, including venous thrombosis [18]. Indel polymorphism in promoter of PAI-1 gene determines presence of two allelic variants: with 4 or 5 guanine repeats (4G/5G), which modify gene expression [19] [20]. 4G variant results in increased expression of PAI-1 and consequently diminished clot degradation, resulting in prothrombotic state [19] [20] [21].

Recently, gene polymorphisms in factor XIII (FXIII) and endothelial protein C receptor (EPCR) have also been studied in association with venous thrombosis and RPL. FXIII covalently cross-links fibrin alpha and gamma chains and plays important role in fibrinolytic system [22] [23] [24]. Most commonly associated with thrombotic events is Val34Leu gene variant in FXIII (V34L) [24]. Its presence leads to enhanced activation of FXIII, enhanced dimerization and polymerization of fibrin chains, which changes the structure of clot, making it more resistant to fibrinolysis [24] [25] [26]. EPCR is key component of protein C anticoagulation system [27] [28]. So far, four receptor haplotypes, determined by presence of 13 single nucleotide polymorphisms in linkage disequilibrium, have been described (H1 to H4) [28]. H1 haplotype, tagged with minor allele G4678C, is associated with elevated levels of APC and low risk for clot formation, while H3 haplotype (A4600A) predisposes to thrombosis [27] [28] [29].

Although described gene variants and their link to adverse pregnancy outcome, including RLP, were subject of numerous studies, the results are still conflicting. The aim of the present study was to compare the frequency of FII G20210A, FVL, FVR2, MTHFR C677T and A1298C, PAI-1 4G/5G, FXIII V34L gene variants and EPCR haplotypes in series of patients with RPL with control group. This is the first study in which prevalence of FVR2, MTHFR A1298C, FXIII V34L and EPCR haplotypes was investigated in Serbian population.

## Materials and Methods

Study was designed as prospective case control study. It was conducted at Gynecology and obstetrics Clinic »Narodni front«, Belgrade from 2014. to 2016. Study group was comprised of 70 women experiencing 3 or more consecutive pregnancy losses. Thirtyone age-matched women with 2 or more successful pregnancies and no pregnancy losses were selected as control group. Exclusion criteria for the study were: anatomic abnormalities of uterus, acquired thrombophilia, abnormal peripheral blood karyotype, urogenital infective diseases and endocrinologic disorders. The study was conducted in accordance with Declaration of Helsinki and with approval of local Ethics committee.

Peripheral blood was taken on EDTA as anticoagulant. Genomic DNA was extracted using Thermo-Fisher Pure link kit. Nine thrombophilic gene variants (FII G20210A, FVL, FVR2, MTHFR C677T and A1298C, PAI-1 4G/5G, FXIII V34L and EPCR haplotypes H1 and H3) were simultaneously amplified in single multiplex amplification reaction (Vienna lab StripAssay, Vienna, Austria) as described previously [30]. Reverse hybridization of amplified DNA fragments to test strips was done, as well as their visualization by use of streptavidin-alkaline phosphatase conjugate and color substrates.

Statistical analysis was performed using Statistica 6.0 and SNPstats programs. Age differences in examined groups were tested by Student's t-test. Hardy-Weinberg equilibrium was assessed by chisquare. The prevalence of gene variants in study groups was done by Fisher's exact test. Odds ratio (OR) and 95% confidence intervals (95%CI) were calculated by using logistic regression. Haplotype frequencies and association with outcome were determined by SNPstats software. P values less than 0.05 were considered statistically significant.

## Results

Recurrent pregnancy loss (RPL) group and control group (CG) were age matched (mean age 33.2±5.4 v.s. 33.2±4.7, P=0.831). All tested gene variants were in Hardy-Weinberg equilibrium, except for MTHFR A1298C in control group (P=0.012).

Significant difference in genotype frequencies among tested groups was observed only for MTHFR A1298C (P=0.010, Table 1). Although in RPL group prevalence of FV Leiden of FII G20210A gene variants was 11.4% compared to 0.0% in control group, the differences did not reach significance (Table 1).

**Table 1:** Allele frequencies (%) of analysed gene variants (FVL, FVR2, FII G20210A, MTHFR C677T and A1298C, PAI-1 4G/5G, FXIII V34L) in recurrent pregnancy loss and control group (Wt-wild type, Hz-heterozygous Ho-homozygous)

		RPL N=70			CG N=31		
Genotypes	Wt,%	Hz,%	Ho,%	Wt,%	Hz,%	Ho,%	P
FV Leiden	88.5	11.4	0.0	100.0	0.0	0.0	0.102
FVR2	67.1	32.8	0.0	70.7	29.3	0.0	0.818
FII	88.5	11.4	0.0	100.0	0.0	0.0	0.102
MTHFR C677T	50.0	42.8	7.1	38.7	58.1	3.2	0.411
MTHFR	48.5	35.7	15.7	39.7	61.3	0.0	0.010
FXIII V34L	42.8	52.8	4.3	67.7	32.3	0.0	0.064
PAI-1 4G/5G	18.5	47.1	34.3	35.0	58.1	35.5	0.283

**Figure 1:**
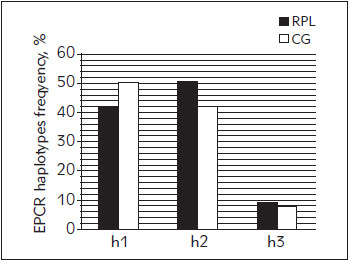
EPCR haplotype frequencies (%) in examined groups

**Figure 2:**
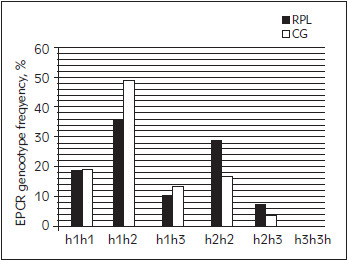
EPCR genotype frequencies (%) in tested groups

Results of EPRC haplotype and genotype frequencies are shown in Figure 1 and Figure 2, respectively.

The most frequent EPCR haplotype in RPL group is h2 (50%), and in control group h1 (50%) (Figure 1). H3 haplotype has similar frequency in both groups (8.6% vs. 8.1%, Figure 1). Most common EPCR genotype in both groups is h1h2 (25 vs. 15%), while h3h3 genotype wasn't detected in tested subjects (Figure 2). There was no significant difference in frequency of EPCR haplotypes or genotypes in tested groups (P=0.521 and P=0.642).

Statistically significant association with recurrent pregnancy loss was determined for FXIII V34L gene variant (P=0.023, Table 2). Carriers of V34L have 2.81 times higher risk for RPL (OR 2.81, 95%CI 1.15-6.87, Table 2). Other examined gene variants didn't show significant association with adverse pregnancy outcome Table 2.

**Table 2:** Association between tested genotypes and recurrent pregnancy loss (dominant genetic model was used) OR – odds ratio; CI – confidence interval; NA – not estimated; A – adenine; G – guanine; T – thymine; C – cytosine

Genotypes	OR (95% CI)	P
FV Leiden GG+GA vs. AA	NA	NA
FVR2 AA+AG vs. GG	1.2 (0.48–3.03)	0.700
FII GG+GA vs. AA	NA	NA
MTHFR C677T CC+CT vs. TT	0.63 (0.27–1.49)	0.291
MTHFR A1298C AA+AC vs. CC	0.58 (0.24–1.39)	0.224
FXIII GG+GT vs. TT	2.81 (1.15–6.87)	0.023
PAI-1 5G5G+4G5G vs. 4G4G	1.05 (0.43–2.58)	0.910
EPCR AA+AG vs. GG	1.07 (0.33–3.67)	0.900

Next, we examined association of thrombophilic gene haplotypes and RPL. Results are shown in Table 3. Association with recurrent pregnancy loss was significant for GAGCATGA haplotype (haplotype with compound heterozygosity for FXIII V34L and PAI-1 4G, P=0.044, Table 3). Combined presence of FXIII V34L and PAI-1 4G gene variants increases the odds for recurrent pregnancy loss 13.98 times (OR 13.98, 95%CI 1.11–17.46, Table 3). Other haplotypes showed no significant association with RPL (Table 3).

**Table 3:** Association between investigated thrombophilic haplotypes and recurrent pregnancy loss OR-odds ratio; CI-confidence interval; A-adenine; G-guanine; T-thymine; C-cytosine; WT-wild type; Mut-mutant allele. Nucleotides corresponding to »wild type« and »mutant alleles« of tested genes variants are shown in legend below. Table 4

Haplotype	FVL	FVR2	FII G21210A	MTHFR C677T	MTHFR A1298C	FXIII V34L	PAI-1 4G/5G	EPRC	Haplotype frequency	OR (95% CI)	P
1	G	A	G	C	C	G	G	A	0.1732	1	–
2	G	A	G	C	A	G	G	A	0.1347	0.64	0.560
3	G	A	G	C	A	G	GG	A	0.0999	0.99	0.991
4	G	A	G	C	A	T	G	A	0.0873	13.98	0.044
5	G	A	G	T	A	G	GG	A	0.0841	0.99	0.992
6	G	A	G	T	A	G	G	A	0.0648	0.58	0.563
7	G	A	G	T	A	T	GG	A	0.0553	2.21	0.501
8	G	A	G	C	C	T	GG	A	0.0365	2.52	0.523
9	G	A	G	T	A	T	G	A	0.0297	0.24	0.442
10	G	G	G	C	A	G	GG	A	0.028	0.15	0.176

**Table 4:** 

	FVL	FVR2	FII G21210A	MTHFR C677T	MTHFR A1298C	FXIII V34L	PAI-1 4G/5G	EPRC
WT	G	A	G	C	A	G	GG	A
Mut	A	G	A	T	C	T	G	G
								

## Discussion

Well-balanced maternal haemostatic response is necessary for successful pregnancy outcome . Therefore, numerous studies suggested increased prevalence of prothrombotic mutations in [31] women experiencing pregnancy complications such as RPL [32] [33]. In this study we evaluated the influence of nine thrombophic gene variants on recurrent pregnancy loss.

Our results show that prevalence of nine thrombophilic gene variants is not significantly increased in group of women with RPL (Table 1).

Several studies found that FV Leiden and FII G20210A gene variants are major risk factor for recurrent miscarriage [10] [34] [35] [36] [37] [38] [39]. Other studies, however found weak or no association of mentioned mutations and RPL [4] [40] [41]. Results of multi-centric EPCOT study have shown that risk of early miscarriage is not increased in carriers of FV Leiden mutation, and that it increases the risk for late fetal loss [40]. Retrospective studies established association between FII mutation and RPL [42] [43] [44], but numerous prospective have failed to confirm such a connection [45] [46] [47] [48] [49]. Although relative risk for pregnancy loss is 2-fold increased in FII G20210A carriers, absolute risk for adverse outcome remains low and additional risk factors are required for such complication to develop [12]. We found no association between FVL, FII and RPL, which can be due to small sample size and study design itself (Table 2).

Few studies have analysed association of FVR2 gene variant and RPL [50] [51] [52]. They concluded that FVR2 doesn't represent risk factor for RPL, which is in concordance with our results [50] [51] [52]. Frequency of FVR2 heterozygotes in published studies ranged from 3.6-18% in controls and from 6.8-16% in women with RPL [50] [51] [52]. We found higher prevalence of heterozygous carriers of FVR2 in our study group (29% and 32%, Table 1), which can be explained by different ethnical background of investigated populations.

Although recent meta-analysis suggest association of MTHFR gene variants and adverse pregnancy outcomes, those results should be interpreted carefully since most of them are based on results from Asian population and no other possible causes of RPL were taken into account [53]. It is well known that frequency of polymorphic alleles varies among populations of different ethnic background. We found no significant association of MTHFR C677T and A1298C and pregnancy loss (Table 2), similar to other studies [50] [51] [52] [54] [55]. Our result showed significant difference in A1298C allele frequencies among tested groups (P=0.010, Table 1) with increased number of A1298C heterozygote carriers in control group. Relatively small CG sample size and deviation from HV equilibrium in CG for variant in question can be possible explanation for these results. Homozigosity for C667T or compound heterozygosity of C677T and A1298C, if homocysteine serum levels are normal, represent no major risk factor for adverse pregnancy outcomes [56]. There is growing evidence that MTHFR testing has minimal clinical utility, thus American college of medical genetics (ACMG) recommends that it should not be ordered as a part of routine evaluation for thrombophilia [56].

Increasing evidence supports role of EPCR h3 haplotype as a risk factor for thrombotic events [27] [28] [29]. H3 haplotype carriers have increased levels of soluble EPCR (sEPCR), decreased levels of functional membrane-bound EPCR, and thus reduced rate of PC activation [27] [28] [29]. Meta-analysis of Dennis and coworkers [27] showed that h3 haplotype frequency among healthy subjects can differ significantly in various populations, but that it ranges form 10-31%, for h3 heterozygotes and from 0-5% for h3 homozygotes. Frequency of h3 haplotypes in our control population matches published results (16% for h3 heterozygotes and 0% for h3 homozygotes, Figure 1). Animal studies have shown that *PROCR* gene coding for EPCR is necessary for early embryonic development [57] [58]. PROCR knock out mice show early embryonic lethality (before day 10.5). However, if such embryos are separated from extra-embryonic structures, they survive in vitro, which implies crucial role of *PROCR* gene in proper placental development [58]. EPCR is expressed on surface of giant trophoblast cells and is in direct contact with maternal circulation. Extra-embryonic cells lacking surface EPCR are surrounded by fibrin deposits and clots, further supporting the important role of this receptor in controlling of coagulation processes on maternalfoetal interface [58]. Different findings regarding EPCR haplotypes and adverse pregnancy outcome have been reported. Dendana et al. [59] showed that risk for RPL is increased in carriers of h3 haplotype. Cochery-Nouvillon et al. [60] concluded that if fetus has h3h3 genotype, risk for miscarriage is further increased. However, we found no significant association between EPCR haplotypes and recurrent miscarriage nor significant difference among haplotype frequencies in tested groups (Table 2, Figure 1). Hopmeier et al. [29] suggested protective role of h1 haplotype against RPL, especially in FVL mutation carriers, while they found no significant change in relative risk for RPL in h3 haplotype carriers, and assumed that influence of EPCR haplotypes on RPL risk is small. Similar results are obtained by Kaare et al. [57], which concluded that mutations in EPCR are not considered to be major risk factor for recurrent miscarriage. However, additional prospective studies are needed to further investigate this link.

Prevalence of heterozygotes for FXIII V34L gene variant in our study (32% for CG and 53% RPL group, Table 1) matches previously published data in European and North American populations [61] [62]. Although we found no difference in frequency of FXIII V34L allele among tested groups (P=0.064, Table 1), risk for miscarriage in carriers of V34L is increased 2.81 times (OR 2.81, 95 %CI 1.15–6.87, P=0.023, Table 2). Several studies are concordant with our results [50] [51] [52].

Frequency of PAI-1 4G/4G gene variant was not increased among women experiencing RPL compared to control, nor it was associated with increased risk for RPL (Table 1 and Table 2). Djordjevic et al. [19] reported that PAI-1 4G/4G doesn't confer increased risk for early foetal loss. Similar results are published by various studies [50] [51] [52], although some reports suggest otherwise [63].

Interestingly, our result show that combined presence of XIII V34L and PAI-1 4G gene variants leads to substantial increase in risk for RPL (OR 13.98, 95%CI 1.11-17.46, Table 3). Study by Dossenbach et al. concluded that isolated presence of FXIII V34L or PAI-1 4G variants represents no risk factor for RPL, but if present in combination it significantly increases the risk or RPL [62]. These observations have a sound pathophysiological explanation. Successful placentation depends on adequate throphoblast invasion in endometrial tissue and its stabilisation by forming of fibrin links. V34L gene variant changes the structure and quality of blood cloths. Accelerated fibrin formation caused by V34L presence inhibits lateral aggregation of fibrin fibers, which reduces mass/length ratio. Newly formed fibrin has finer mesh structure with thinner fibers that are more densely placed. This leads to reduced fibrinolytic activity, since t-PA and u-PA perform better on coarse fibrin mesh with larger pores [24] [62] [64]. Hypofibrinolysis caused by increased PAI-1 expression in 4G variant carriers can lead to fibrin over-deposition and consequent disruption of trophoblast migration during early stages of placentation [21] [22]. Our data suggest that V34L and PAI-1 4G may have additive effect by increasing fibrin resistance to degradation and reducing the activity of fibrinolytic system, thus leading to impaired placentation.

In conclusion, this is the first study in Serbian population that investigated prevalence of FVR2, A1298C, FXIII V34L and EPCR gene variants. Our data shows that compound heterozygosity for FXIII V34L and PAI-1 4G is significant risk factor for recurrent miscarriage. As polymorphisms in FXIII are currently not part of routine thrombophilia testing panel, we suggest that it should be included in diagnostic testing as it can contribute to more precise risk estimation for RPL. Although our result should be viewed in context of small case-control study, tested population was highly selected, all other known causes of pregnancy loss were previously excluded in patients. Nevertheless, further large prospective studies are need for confirmation of our findings.

*Acknowledgements*. This work was supported by the project funded by Ministry of Education, Science and Technological Development of the Republic of Serbia (project No. ON173046)

### Conflict of interest statement

The authors stated that they have no conflicts of interest regarding the publication of this article.

## List of abbreviations

RPL, recurrent pregnancy loss; FV, FactorV; prothrombin, FII; MTHFR, methylene tetrahydrofolatereductase; APC, activated protein C; FXIII, factor XIII; EPCR, endothelial protein C receptor; PAI-1, plasminogen activatorinhibitor-1; CG, control group; t-PA, tissue-type plasminogenactivator; u-PA, urokinase-type plasminogen activator.
